# Infective Diseases

**Published:** 1895-03-16

**Authors:** 


### PROGRESS IN MEDICINE.

### INFECTIVE DISEASES.

(*Continued from page* 404).

**Immunity** is ascribed either to the action of the fluids or to the cells of the body. Buchner is the representative of one school, Metchnikoff of the other; but considerable approximation has taken place between them. Thus Buchnerl now states that natural immunity is caused by dissolved alexines, and the resistance of the tissues, while leucocytes act by their secretions, not chiefly by phagocytosis. Acquired immunity is due to the presence of modified bacterial products, which increase the resisting power of the tissues, but are not themselves destructive of the poisons. Roux points out[2] that the serum of animals vaccinated against cholera, pneumonia, &c., has no anti-toxic powers, though it protects against and destroys the microbe, but not its toxin, while in tetanus, diphtheria, &c., the serum is destructive to the special toxin. He holds, too, that the anti-toxin of the serum is not derived from the toxin, but from the cells of the host, and he agrees with Buchner that it acts by increasing their resisting power. This action may extend to more than one species of toxin. Kanthack and others suggested that the eosinophilous cells secrete the alexines, but Metchnikoff points out that certain fishes which have no such cells destroy microbes just as well. Leucocytosis occurs when non-fatal doses of toxins are injected, and in animals habituated to arsenious acid. Moreover, Foder[3] finds that in similar circumstances the alkalinity of the blood increases in relation the degree of resistance the animal possesses. Pagano tries to show that while serum has bactericidal powers, lymph has none, and is even a good culture medium.[4] Suppuration can be produced by dilute croton oil without the presence of microbes, while stronger solutions produce necrosis, according to Durochowski.[5]

**Antipyretics.**—Much discussion has gone on respecting the effect of painting guiacol on the skin. Guimard[6] finds that sparteine has even greater effects which may be permanent in diseases where the skin is affected, as in erysipelas and measles. Ferrand and others[7] say that the antithermic effects of guiacol are greater if applied pure, and the analagesic effects better if mixed with glycerine. They report highly of its value against the neuritis and other pains of consumptives. B. W. Richardson, speaking of the properties of a good antipyretic[8], lays down that it should be antiseptic, volatile, and not too soluble in the blood, and finds that chloral hydrate fulfils these conditions, and, when properly administered, is an admirable remedy for pyrexia. A report on acetanilide confirms the belief in its efficacy, but emphasises the risk of untoward effects from its use.[9] Phenacetin appears to be much safer, though undue sweating and collapse may follow even moderately large doses. If impure, it may cause kidney troubles. In fact, it is doubtful whether any of these new drugs diminish the production of heat, though they increase its dispersion. Quinine alone has the power of checking thermogenesis, and remains our most powerful antipyretic.

**The Plague.**—Kitasato's report shows that the bacilli occur in the blood, the glands, and even in the intestinal tract; they grow best on blood serum, which they do not liquefy. Mice, rats, and rabbits are very susceptible. Bright sunshine destroys the microbes in a few hours, boiling in a few minutes, and four days' drying has the same effect. One per cent. of quicklime or carbolic acid are fatal. He considers that[10] they enter either by the respiration, through a wound, or by the food, and it is remarkable that no other bacilli except those of anthrax and relapsing fever have been found in the blood. The disease appears to be permanently endemic in Yunnan.

**Malta Fever.**—Hughes states that the bacillus is distinct from that of typhoid. In obstinate cases the fever may last six months, more often it lasts only two or three weeks with apyretic intervals. There is prolonged constipation, anæmia, and great debility, sometimes bronchitis and hyperpyrexia; the mortality is 2 per cent.[11] Quinine and arsenic are useless. Burke advocates[12] naphthol and enemata for the bowels as well as purgatives and liquid food. Change of climate seems to be at present the chief remedy.

**Scarlet Fever.**—Hodges[13] reviews 117 cases of rheumatism complicating scarlet fever in 3,026 cases of the latter affection. It is essentially the same as ordinary rheumatism, and generally arises about the end of the first week. Delirium frequently ushers in the attack, and should lead to one being on the outlook for it. The complication affects much the same joints as in cases without scarlet fever, and is most frequent in older children and young adults. Erythema urticaria and purpura were seen, but no case of suppurative arthritis occurred out of 7,000 scarlatina patients in three years. There appeared no connection with septic cases, nor with ulceration of the throat. Heart complications were noted in 24·7 per cent., excluding doubtful cases. Hodges notices that a patient who has had previous rheumatism almost always develops arthritis during scarlet fever, but it adds little of itself to the fatality of that disease. W. A. Parker refers to 53 cases[14] occurring in 870 of scarlet fever, one of them as late as the eighth week. His conclusions are much the same as those of Hodges, both with regard to the similarity to ordinary rheumatism and to other points. He notes four cases of suppurative arthritis from pyæmia, but none of them followed rheumatism. One case of ulcerative endocarditis developed, and ended in recovery. Both sets of statistics show the occasional presence of chorea. In connection with the occasional urticaria in these rheumatic patients, Coulon reports[15] an instance where severe urticaria took the place for a time of the typical scarlatinal rash, without any later manifestation of rheumatism. Sacaze looks on the injured tonsils as affording an entrance to staphylococci of feeble virulence, and he quotes[16] authorities as showing that they have power to cause effusions in the joints. Thus Bouchard and Charin in 1891 found the staphyloccus many times in the effusions of ordinary rheumatism.[17] Sacaze believes that some wound or place of entry can often be found preceding attacks of rheumatism. Rocaz reports a case apparently of scarlatina with rheumatism, in which pus was formed near, but not in, the joints and which was found to contain streptococci.[18] Perforation of the soft palate in ordinary scarlatina was seen fourteen times by G. W. Goodall, but no special importance seems to be attached to it.

**Tetanus.**—A large number of cases treated by serum have been reported. The two first successful ones in this country were those of Mr. Herbert Evans, of Goring, and Mr. Dean.[20] Both these, however, were instances in which the symptoms developed some time after infection. In such cases the course is usually more benign. The same may be said of eleven successful cases previously reported by Tizzoni and Cattani, where the effect of the serum is really doubtful. Six more acute attacks were treated by Behring's serum, of which four survived;[21] but whether a larger proportion would have died under other measures is by no means clear. It is easy enough to produce immunity for a time, but the power of the serum to stop an existing severe case of tetanus is another matter. Rotter's patient developed fairly severe symptoms on the eighth day after infection, and recovered rapidly under large repeated doses, amounting to 250 grammes of serum.[22] Guisti reports a case of some value where trismus followed on the day after infection, probably from the presence of formed toxin in the earth. No other symptoms occurred for twenty days, when a most severe attack with cardiac symptoms and vomiting came on. After three days' trial of other measures, Tizzoni gave large repeated injections of horse and dog serum, and in three days all symptoms disappeared.[23] In spite of the severe type, however, the prognosis could not have been of the most unfavourable kind after so long a period of quiescence had occurred. A patient in Northampton, under the care of John Marriott and Buszard, manifested symptoms on the sixth day, which gradually increased; anti-toxin was administered on the twelfth day and subsequently with success. On two occasions, when very severe spasms threatened to put an end to life, morphia and gr. $\frac{1}{50}$ of physostigmine were given, and produced temporary improvement. The second severe attack followed a stoppage of the anti-toxin. The serum was given for a fortnight, and improvement and steady progress, with complete recovery, was made. This seems to be one of the most complete and satisfactory cases which have been reported. The value also of the physostigmine and morphia in the sudden emergencies was very great, and apparently prevented the patient's death at the time. An unsuccessful case of treatment of tetanus neonatorum is reported by L. Firth from the General Hospital, Bristol. The disease developed on the eighth day, and anti-toxin was injected after a week of the illness for three days without improvement. An interesting feature in the illness was the occurrence of complete asphyxia, with stoppage of respiration, followed by relaxation of the muscular spasms, though respiration did not return on one occasion for seven minutes. Bauer, at Vienna, reports a case where symptoms appeared on the seventh day with great severity. Chloral and one dose of 2·25 grammes of anti-toxin were given without effect, death occurring on the tenth day.[25]

The immense expense of the serum is a serious difficulty. Two patients in America consumed as much as £18 worth. G. Thompson describes the first case treated in that country from a toxin made after Brieger's method. The symptoms had commenced fourteen days after the wound, and the patient seemed to be gradually failing when the anti-toxin was used. Complete recovery followed, but the author expresses his own doubt as to its real effect.[26] Doebler reports a[27] case of moderate severity; Rushton Parker one of chronic tetanus, which improved rapidly under injections; and C. E. Douglas[28] a fatal case where the symptoms began on the fifth day, and only one full dose of anti-toxin was injected. He notes the difficulty of quickly dissolving the dried serum. E. Schwartz gives another unsuccessful case, with temporary improvement, great leucocytosis, but death from heart failure.[29] He concludes that stronger doses ought to be employed, since he found that the first dose of $2\frac{1}{2}$ grammes reduced the excessive urinary elimination of phosphorus, while the half-gramme doses given afterwards had no such effect. Von Hacker's two cases were successful. He describes the prognoses in both as bad, but the second was certainly not of an acute type.[30] So, too, with Tizzoni's last case, where treatment commenced twenty-four days after the injury.[31] On the whole, in spite of the favourable results in many cases, and especially in the acute one reported by J. Marriott, it will be agreed that we must see the results in a much greater number before anything like a definite verdict on the value of the treatment can be pronounced. However, it appears so far harmless, and may possibly be less absurdly expensive before long.

[1] Amer. Jour. Med. Sci., Jan., 1895. [2] Med. Week, Sept. 14. [3] Ibid. [4] Brit. Med. Jour., Dec. 1. [5] Presse Med., Nov. 10. [6] Lancet, Aug. 23. [7] Amer. Jour. Med. Sci., Oct. [8] Asclep, No. 41. [9] Brit. Med. Jour, Dec. 22. [10] Pract., Oct. [11] Amer. Jour. Med. Sci., Sept. [12] Brit. Med. Jour., Sept. 23. [13] Lancet, Nov. 17. [14] Lancet, Dec. 1. [15] Amer. Jour. Med. Sci. [16] Brit. Med. Journ., Dec. 1. [17] Med. Rec., Dec. 1. [18] Brit. Med. Journ, Dec. 1. [19] Lancet, Dec. [20] Brit. Med. Journ., Sept. 22. [21] Therap. Gaz., Feb., 1894. [22] Med. Rec., June 9. [23] Brit. Med. Journ., June 16. [24] Brit Med. Journ., Jan. 19. [25] Brit. Med. Journ., Dec. 22. [26] Med. Rec., Jan. 6; Amer. Journ. Med. Sci., Nov. [27] Brit. Med. Journ., Nov. 24. [28] Brit. Med. Journ, Oct. 27. [29] Med. Week, Nov. 9. [30] Amer. Jour. Med. Sci., Dec. [31] Amer. Jour. Med. Sci., Dec.

